# Consecutive bilateral breast reconstruction using different autologous flaps: can symmetrical results still be achieved? A case report

**DOI:** 10.1080/23320885.2023.2197500

**Published:** 2023-06-27

**Authors:** Antonios Tsimponis, Dimitrios Dionyssiou, Spyros Miliaras, Efterpi Demiri

**Affiliations:** a Department of Plastic Surgery, Aristotle University of Thessaloniki, Thessaloniki, Greece; b 1st Department of Surgery, School of Medicine, Aristotle University of Thessaloniki, Thessaloniki, Greece

**Keywords:** Autologous breast reconstruction, deep inferior epigastric perforator flap, fat-augmented latissimus dorsi flap

## Abstract

We report a 60-year-old patient who underwent bilateral mastectomy at different times, followed by immediate autologous reconstruction with different flaps: deep-inferior epigastric-perforator flap on one breast, and fat-augmented latissimus dorsi on the contralateral side. At 20-month follow-up, good symmetry was recorded; patient-reported outcome measurements revealed high satisfaction scores.

## Introduction

Post-mastectomy autologous breast reconstruction is a demanding procedure aiming to rebuild a complex three-dimensional breast mount with autogenous tissues. The degree of difficulty might be greater in bilateral breast reconstructions when a symmetrical outcome is needed; however, post-reconstruction breast asymmetries are commonly encountered [1]. Among the major factors that contribute to non-symmetrical results are the different reconstructive methods that may be used (i.e. unilateral use of implants [2] or different flaps used in autologous breast reconstruction [3], the timing of reconstruction following mastectomy [4], previous treatments performed in each breast, and/or the use of radiation [5,6].

In women who undergo bilateral autologous breast reconstruction, the use of two flaps of the same size and identical characteristics, cannot always be achieved, especially in patients who receive a contralateral prophylactic or therapeutic mastectomy, months or years following the first mastectomy. Moreover, in women who already had an abdominal-based autologous breast reconstruction, the number of available flaps suitable for a symmetric contralateral result is limited.

In this paper, we report a case of bilateral mastectomy performed at different times on each breast, followed by immediate autologous breast reconstruction with two different flaps, which ended in a highly symmetrical result.

## Case presentation

The case report presented has been performed in accordance with the principles stated in the Declaration of Helsinki and approved by the ethics committee of Aristotle University of Thessaloniki (reference nb 1456/23.12.2022) and the subject provided fully informed consent.

**Figure 1.**
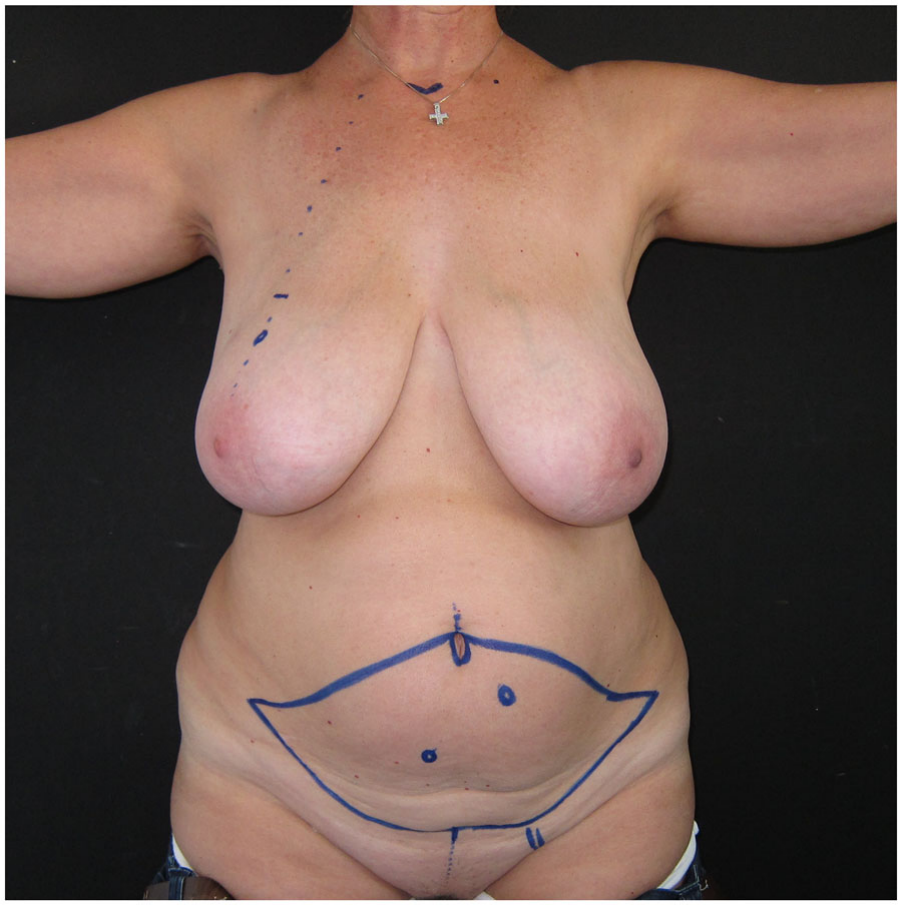
Pre-operative drawings of the skin island of the DIEP flap and the selected left-side perforator, for immediate reconstruction following the planned right breast skin-sparing mastectomy.

A 60-year-old woman was presented to our breast clinic after being diagnosed with recurrent ductal cancer of her right breast. She had a history of right-breast tumorectomy followed by radiotherapy seven years ago, and a right skin-sparing mastectomy was planned by the breast surgeon. The patient had a negative family history of breast cancer, and had not been tested for hereditary breast and ovarian cancer. At clinical examination, the patient presented large, almost symmetrical breasts, with grade C ptosis; local lipodystrophy and laxity of the abdominal tissues were recorded. The patient had two pregnancies and a Body Mass Index of 28,2kg/m2 (Figure 1). According to her medical history, no other comorbidities were reported.

**Figure 2.**
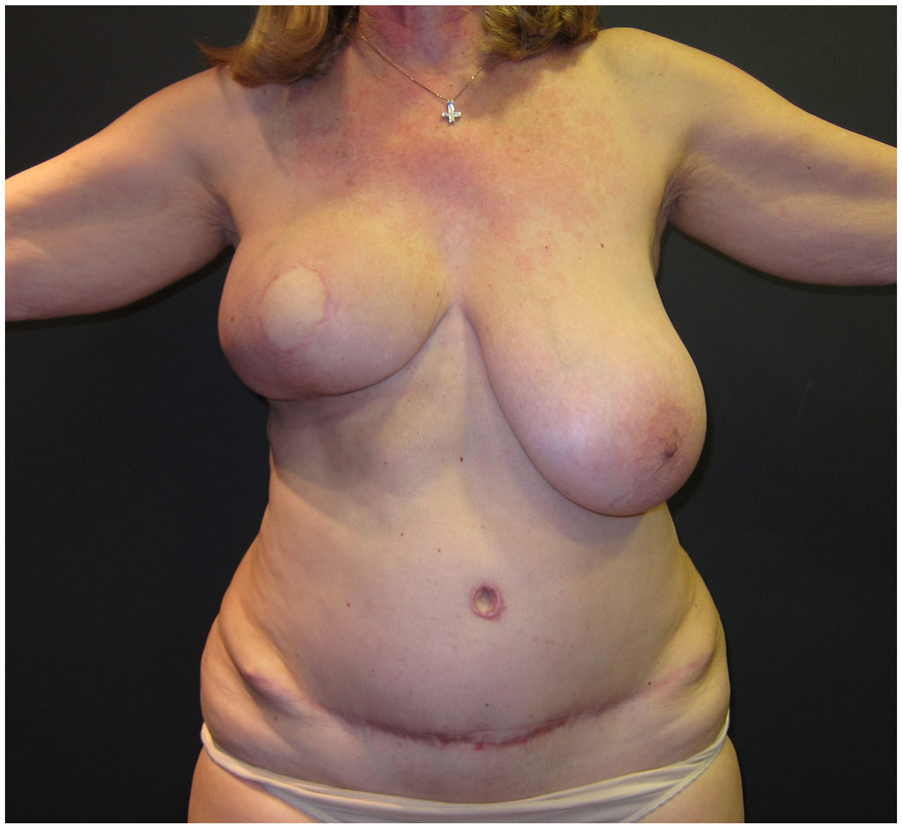
Post-operative view at 6 months following the immediate DIEP flap-based right breast reconstruction; a significant asymmetry is Present, as no symmetrisation surgery was performed on the left breast.

A skin-sparing right mastectomy was performed according to the Wise-pattern skin excision, including the nipple-areola complex; mastectomy was followed by immediate breast reconstruction with a deep-inferior epigastric perforator (DIEP) flap, with exteriorization of a skin island of the flap to reconstruct the areola and monitor the flap’s viability. The postoperative course was uneventful; the patient was discharged on the seventh day after surgery. As the patient did not wish to have any symmetrization procedure on her left healthy breast, a post-operative breast asymmetry was obviously expected (Figure 2).

One year later, the patient was diagnosed with *in situ* breast cancer on the contralateral breast, and a plan for a left skin-sparing mastectomy was set. As the patient did not wish to have a second microsurgical procedure, nor an implant-based reconstruction, an autologous immediate breast reconstruction with a pedicled latissimus dorsi myo-cutaneous flap augmented with fat was suggested and discussed with the patient.

**Figure 3.**
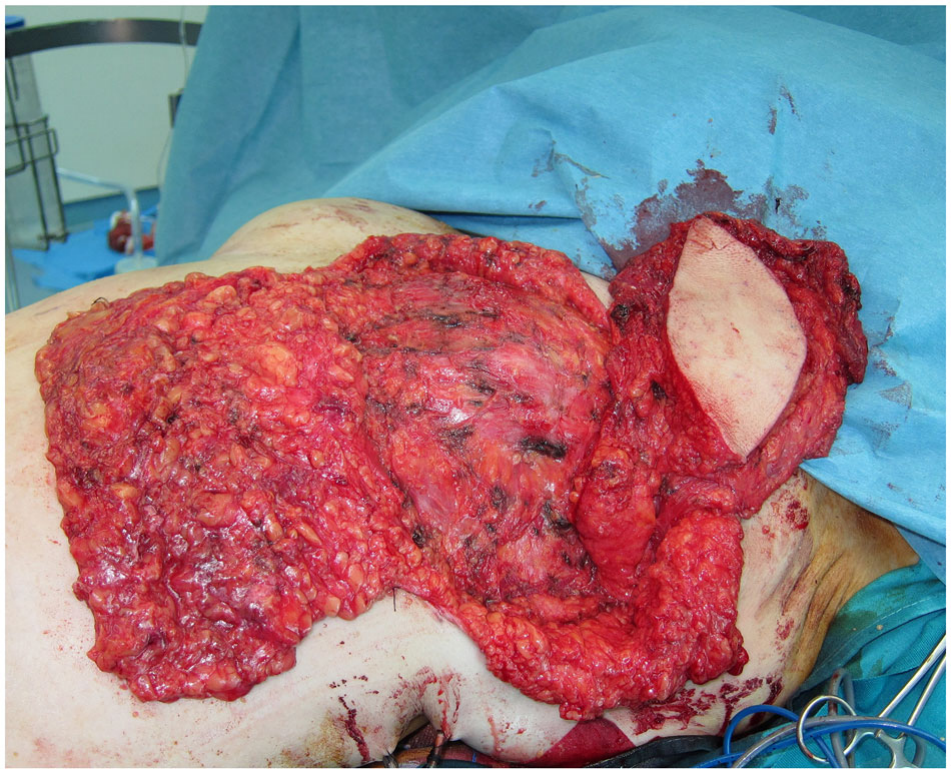
Intraoperative view of the fat-augmented latissimus dorsi (FALD) flap after the fat injections into the LD flap, the left pectoralis major muscle, the mastectomy skin flaps and the de-epithelialized dermo-fatty pedicle of the lower breast pole.

Following a Wise-pattern skin-sparing left mastectomy including the nipple-areola complex, a tumescent liposuction was performed over both the lateral lumbar areas and the thighs, and 600 ml of lipoaspirate was removed. A myo-cutaneous latissimus dorsi (LD) flap with a skin island measuring 12 × 6 cm, was elevated and transposed to the anterior thorax through a sub-cutaneous axillary tunnel (Figure 3). Sub-Scarpa fat above and below the skin paddle was included in the flap, in a tilted fashion, for additional bulk. Care was taken to avoid traumatizing the fascia, while numeral quilting sutures were placed at the donor site to avoid seroma formation. The lipoaspirate was prepared, washed, and filtered, and a total of 300 ml fat was injected in an axial pattern into the pectoralis major and the latissimus dorsi muscles, and into the subcutaneous tissues of the flap’s skin island, the mastectomy flaps, and the de-epithelialized dermo-fatty pedicle of the lower breast pole. The fat-augmented latissimus dorsi flap was folded, sutured on the thoracic tissues, and wrapped by the mastectomy flaps creating a well-projected new breast mount. A round-shaped skin island of the LD flap was exteriorized for a neo-areola reconstruction. The postoperative course was uncomplicated; the patient was discharged after a six-day hospitalization. Ten months later, a second lipofilling session was performed; a total of 200 ml of fat was injected to augment the volume of the left breast.

**Figure 4.**
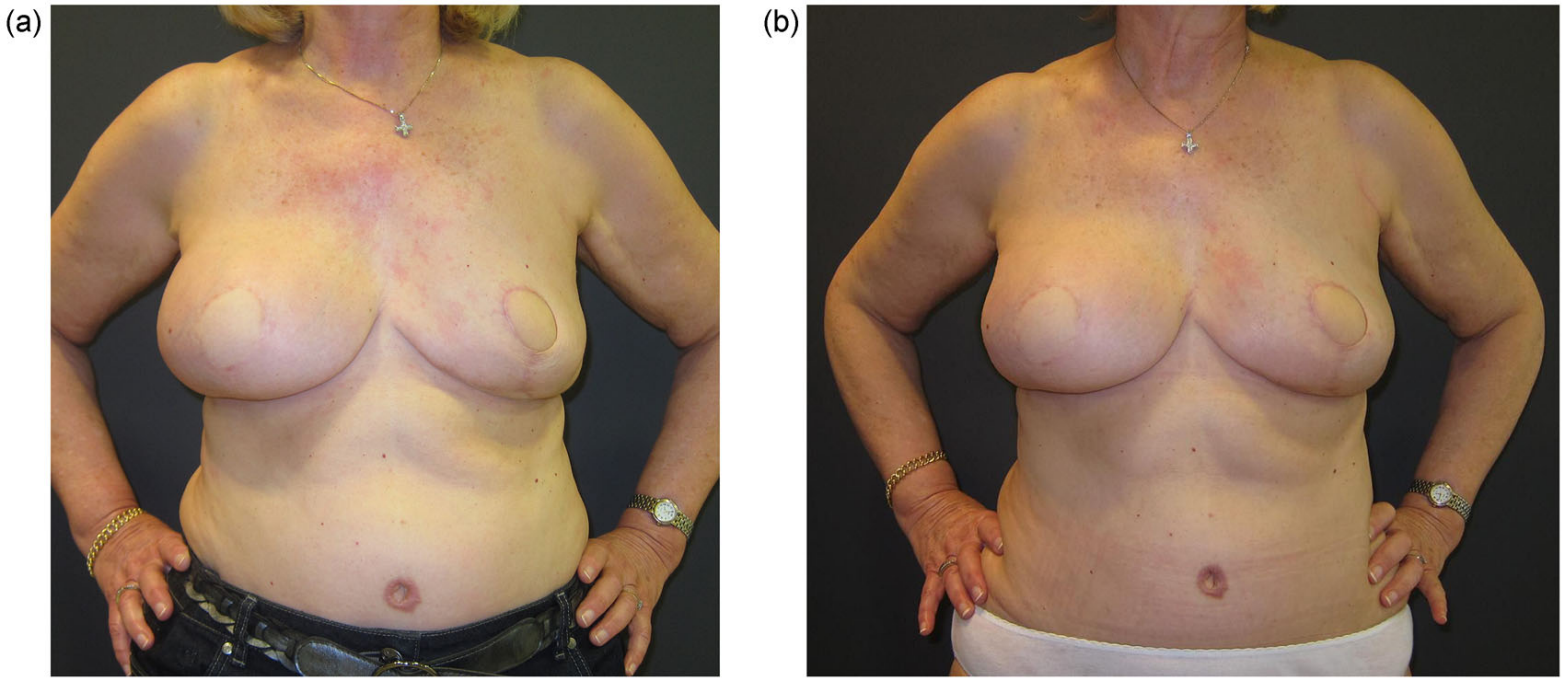
Post-operative result at 10-month follow-up after the left breast reconstruction, when additional lipofilling was Decided for better symmetry between the two reconstructed breasts (a); final post-operative result at 20-month follow-up after the second lipofilling session (b).

The patient was re-evaluated in follow-up visits at six, twelve and twenty months after the second fat-grafting session. Despite the irradiation on the right breast and the different reconstructive approaches, the shape and volume of both reconstructed breasts were comparable, with very similar projection and grade of ptosis. Patient satisfaction was measured through the Breast-Q breast reconstruction module at twenty months (Figure 4) and revealed high scores regarding satisfaction with both breasts.

## Discussion

Bilateral primary breast cancer is not rare, and it is well known that women previously treated for primary unilateral breast cancer are at increased risk of developing metachronous contralateral breast disease, 5.9 times higher compared to the risk of primary breast cancer in the general population [7,8]. Moreover, fat grafts contain progenitor cells and immunomodulatory cytokines, which may interfere with vasculogenesis or tumor progression or recurrence at the site. However clinical research has not showed an increase in breast cancer recurrence after breast fat grafting, except for 1 small study on a patient population with intraepithelial neoplasm of the breast [9].

Symmetry, natural breast appearance, and patient satisfaction are the principal aims of all breast reconstructive procedures [10]. Although bilateral autologous breast reconstructions, when performed simultaneously and using the same flaps, are reported to demonstrate high scores regarding the patient-reported satisfaction [11], in many cases achieving good symmetry consists of a real challenge for the plastic surgeon. Previous breast volumes and shape discrepancies, the immediate or delayed reconstruction type, the flaps that are used for reconstruction, the previous treatments, i.e. surgeries or irradiation, as well as the possible complications that may occur, all comprise independent factors that increase the grade of difficulty to reach a natural and final symmetrical result [1–4].

Various studies have compared the use of different flaps and/or implants in unilateral or bilateral reconstructions and their impact on the final aesthetic outcome, the patient’s satisfaction, and cost-effectiveness [12,13]. Most of them show a preference of both patients and plastic surgeons in immediate breast reconstruction and autologous techniques, especially when irradiation is to be considered [13]; other studies favor implant-based breast reconstruction in selected patients, as the method that may provide significant grade of symmetry [14]. Fat grafting comprises a new addition to breast reconstruction methods [15], having a multi-dimensional role not only in adding volume but also contributing to better contouring and quality improvement of the skin and soft tissues of the reconstructed breast [16]. Most of the time lipofilling has a complementary role [15], although in certain cases it constitutes the main method of reconstruction [17–19].

In the reported case, two different autologous reconstructive methods were applied for immediate breast reconstruction in a bilateral mastectomy patient, who underwent mastectomies at different times: a DIEP was primarily used for the right breast reconstruction, and one year later, a fat-augmented latissimus dorsi myocutaneous flap was transferred for the left breast reconstruction. Flap-selection for the second reconstructive procedure was based on the patient’s wish for an autologous non-microsurgical reconstruction; therefore, a free flap, i.e. SGAP or TUG-flap-based reconstruction was not an option. The patient was pre-operatively informed about possible additional lipofilling sessions in order to get the desired breast volume. Indeed, a secondary lipofilling was performed to add volume to the left LD-based reconstructed breast, ten months following the initial procedure.

The fat-augmented latissimus dorsi myo-cutaneous flap for pure autologous breast reconstruction is yet a well-established procedure [20–23]. In a comparative cohort study between DIEP and LD-based delayed breast reconstructions, comparable outcomes were reported regarding complications and patients’ satisfaction; based on those results, the fat-augmented - LD flap was suggested as a good reconstructive option mainly for thin nulliparous mastectomy patients with small to medium-sized breasts [16].

In the reported case the fat-augmented LD flap is directly compared with the DIEP flap in a multiparous 60-year-old overweight patient with large-sized breasts, who received immediate autologous reconstruction with these two different flaps following mastectomies at different times; interestingly, long-term results showed comparable outcomes in both breasts, with acceptable symmetry and high scores regarding the patient-reported outcome assessment. To our knowledge, a similar comparison of those autologous reconstructive methods in a single patient has not yet been reported.

## Conclusion

In conclusion, LD-based breast reconstruction may provide comparable results and good symmetry to a DIEP reconstruction following mastectomy, in selected cases. It should be considered a reliable autologous reconstructive option when abdominal tissues are not available and/or other flaps are not indicated; additional lipofilling might be needed to achieve the desired breast volume.
